# Synaptic Functions of Hemichannels and Pannexons: A Double-Edged Sword

**DOI:** 10.3389/fnmol.2018.00435

**Published:** 2018-12-04

**Authors:** Verónica Abudara, Mauricio A. Retamal, Rodrigo Del Rio, Juan A. Orellana

**Affiliations:** ^1^Departamento de Fisiología, Facultad de Medicina, Universidad de la República, Montevideo, Uruguay; ^2^Centro de Fisiología Celular e Integrativa, Facultad de Medicina, Clínica Alemana Universidad del Desarrollo, Santiago, Chile; ^3^Department of Cell Physiology and Molecular Biophysics, Center for Membrane Protein Research, Texas Tech University Health Sciences Center, Lubbock, TX, United States; ^4^Programa de Comunicación Celular en Cáncer, Instituto de Ciencias e Innovación en Medicina, Santiago, Chile; ^5^Laboratory of Cardiorespiratory Control, Departamento de Fisiología, Facultad de Ciencias Biológicas, Pontificia Universidad Católica de Chile, Santiago, Chile; ^6^Centro de Envejecimiento y Regeneración, Pontificia Universidad Católica de Chile, Santiago, Chile; ^7^Centro de Excelencia en Biomedicina de Magallanes, Universidad de Magallanes, Punta Arenas, Chile; ^8^Departamento de Neurología, Escuela de Medicina and Centro Interdisciplinario de Neurociencias, Facultad de Medicina, Pontificia Universidad Católica de Chile, Santiago, Chile; ^9^Centro de Investigación y Estudio del Consumo de Alcohol en Adolescentes, Santiago, Chile

**Keywords:** astrocytes, microglia, neuron, LTP, connexin, pannexin, neuroinflammation

## Abstract

The classical view of synapses as the functional contact between presynaptic and postsynaptic neurons has been challenged in recent years by the emerging regulatory role of glial cells. Astrocytes, traditionally considered merely supportive elements are now recognized as active modulators of synaptic transmission and plasticity at the now so-called “tripartite synapse.” In addition, an increasing body of evidence indicates that beyond immune functions microglia also participate in various processes aimed to shape synaptic plasticity. Release of neuroactive compounds of glial origin, -process known as gliotransmission-, constitute a widespread mechanism through which glial cells can either potentiate or reduce the synaptic strength. The prevailing vision states that gliotransmission depends on an intracellular Ca^2+^/exocytotic-mediated release; notwithstanding, growing evidence is pointing at hemichannels (connexons) and pannexin channels (pannexons) as alternative non-vesicular routes for gliotransmitters efflux. In concurrence with this novel concept, both hemichannels and pannexons are known to mediate the transfer of ions and signaling molecules -such as ATP and glutamate- between the cytoplasm and the extracellular milieu. Importantly, recent reports show that glial hemichannels and pannexons are capable to perceive synaptic activity and to respond to it through changes in their functional state. In this article, we will review the current information supporting the “double edge sword” role of hemichannels and pannexons in the function of central and peripheral synapses. At one end, available data support the idea that these channels are chief components of a feedback control mechanism through which gliotransmitters adjust the synaptic gain in either resting or stimulated conditions. At the other end, we will discuss how the excitotoxic release of gliotransmitters and [Ca^2+^]_i_ overload linked to the opening of hemichannels/pannexons might impact cell function and survival in the nervous system.

## Introduction

The traditional view of neurons as the only functional units of synaptic transmission has been challenged in recent decades by the emerging influence of glial cells. Of particular interest for neuroscience research is the modulatory action of glial cells in synaptic transmission, including synaptogenesis, pruning, pre- and post-synaptic maturation and elimination, as well as stabilization of synaptic receptors (Allen and Eroglu, 2017). This is especially relevant for astrocytes, which embody a wide-ranging interconnected entanglement that structurally and functionally establish dynamic and often bidirectional interactions with neuronal synapses (Gundersen et al., 2015). Through its cellular processes, a single astrocyte may contact around 100,000 and 2,000,000 synapses in rodents and humans, respectively (Oberheim et al., 2009). In companion with pre- and postsynaptic neuronal components, astrocytes establish the “tripartite synapse,” a specialized functional structure in where astrocytes sense neurotransmission and respond to it by locally releasing messengers referred to as “gliotransmitters” (e.g., ATP, glutamate and D-serine), which in turn influence synaptic function (Araque et al., 1999; Perea et al., 2009). Additionally, some specialized astrocytes are equipped with unconventional terminal processes termed “endfeet” that contact diverse elements of the vascular system, such as venules, capillaries and intracerebral arterioles (Simard et al., 2003). In this scenario, the resulting astroglial communication with the endothelium and neurons facilitates local and far-reaching signaling of gliotransmitters, vasoactive factors and energy substrates with potentially significant consequences for higher brain functions (Magistretti and Allaman, 2015).

Despite that for long time microglia were considered as worthless elements for synaptic transmission, nowadays they are recognized crucial for a wide range of roles besides their immune function (Morris et al., 2013). In the healthy brain, microglia display a resting surveillance form endowed with dynamic inspection of their territory and continuous scrutinizing for exogenous or endogenous threats (Kettenmann et al., 2011). Along with these features, mounting evidence suggests that microglia continually extend and retract their cell processes toward and from synapses, being part of a new spectrum of unexplored capabilities, such as synaptic pruning, maturation and remodeling, as well as modulation of synaptic transmission and plasticity (Schafer et al., 2013; Wake et al., 2013; Wu et al., 2015). Once microglia detect a disruption in homeostasis, they adopt a reactive phenotype, with a wide degree of activation levels based on nature, intensity and duration of the damage (Li and Barres, 2018). Of note, when severe or chronic brain injury occurs, microglia become activated triggering a widespread release of their inflammatory molecule reservoir, facilitating the engagement of non-resident brain cells implicated in the innate and adaptive immune function (Salter and Stevens, 2017).

**FIGURE 1:**
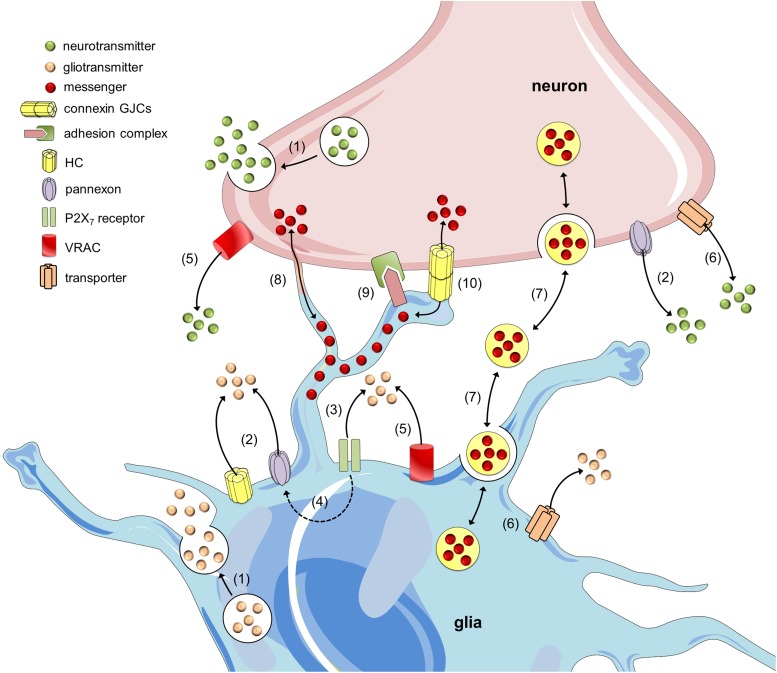
Mechanisms of neuron-glia crosstalk. Glial cells and neurons release neurotransmitters and gliotransmitters through Ca^2+^- and SNARE-dependent exocytosis, respectively (1). In addition to this mechanism, the release of transmitters may occur through alternative non-exocytotic ways. For instance, the opening of hemichannels (HCs) and pannexons may allow the release of gliotransmitters and neurotransmitters (2) (Kang et al., 2008; Xia et al., 2012). Long-lasting activation of P2X_7_ receptors by ATP might lead to the appearance of large currents and the rapid exchange of large molecules, including the release of gliotransmitters (3). One theory states that P2X_7_ receptor conductance dilates over the time and thereby allows the passage of large molecules; however, another hypothesis states that ATP activates a second non-selective permeabilization pathway (Baroja-Mazo et al., 2013). Recently, it was shown that pannexons might mediate this permeability for large molecules in astrocytes (4) (Iglesias et al., 2009). Additionally, gliotransmitter and neurotransmitter release may occur through volume-regulated anion channels (VRAC) (5) (Kimelberg et al., 1990; Fields and Ni, 2010) and different carriers and/or co-transporters acting normally or in reverse (6) (e.g., excitatory amino-acid transporters, the cysteine-glutamate antiporter and the D-serine/chloride co-transporter) (Rossi et al., 2000; Wu et al., 2007). Within the last decade, a growing body of evidence has indicated that glial cells can also communicate with neurons and vice versa via the release of vesicles (e.g., exosomes, microparticles and apoptotic bodies), containing different cellular messengers (e.g., mRNA, viruses and organelles) (7) (Chivet et al., 2012; Fruhbeis et al., 2013). Adjacent glial cells and neurons can communicate directly through F-actin-based transient tubular connections known as tunneling nanotubes (8) (Wang X. et al., 2012), via cell-to-cell contacts between membrane-bound ligand molecules and their receptors (9) (Avalos et al., 2009) or aggregates of intercellular channels known as gap junctions, which allow the exchange of small molecules (10) (Froes et al., 1999).

At the synapse, the communication between neurons and glial cells is bidirectional. Certainly, neurotransmitter release can sculpt multiple facets of glial cell function, such as phagocytosis, cellular migration, Ca^2+^ wave signaling, metabolic cooperation, blood flow regulation, gliotransmission, among others (Stork et al., 2014; Chen et al., 2015; Rosa et al., 2015; Papouin et al., 2017). This reciprocal influence embraces a constant flow of information between neurons and glial cells termed “neuron-glia crosstalk” (Perea et al., 2014). Unlike neurotransmission and despite being a major mechanism underlying neuron-glia crosstalk, gliotransmission has only been studied in recent years. A broad range of pathways have been proposed to sustain gliotransmitter release, such as Ca^2+^-dependent vesicular release (Bezzi et al., 2004; Zhang et al., 2007; Imura et al., 2013), transporters (Rossi et al., 2000) and the opening of several channels. Among the latter group are included P2X_7_ receptors (Duan et al., 2003; Suadicani et al., 2006; Hamilton et al., 2008), volume-regulated anion channels (Kimelberg et al., 1990; Takano et al., 2005; Rudkouskaya et al., 2008), Ca^2+^-dependent Cl^-^ channel bestrophin 1 (Lee et al., 2010; Woo et al., 2012), hemichannels (Stout et al., 2002; Ye et al., 2003; Chever et al., 2014; Meunier et al., 2017) and pannexons (Suadicani et al., 2012; Pan et al., 2015; Garre et al., 2016) (Figure 1). Besides these canonical routes of gliotransmitter release, recent groundbreaking studies have revealed that glial cells communicate with neurons through alternative mechanisms (Figure 1). For instance, heterotypic glia-to-neuron interactions linked to homophilic and heterophilic adhesion molecules control female sexual development and adhesive properties (Avalos et al., 2009; Sandau et al., 2011). In the same line, extracellular exosomes, microparticles or apoptotic bodies allow the transfer of gliotransmitters, organelles, DNA/RNA, proteins and pathogens between glial cells and neurons (Fruhbeis et al., 2013). At the other end, direct glia-to-neuron signaling not only takes place via gap junctions (Froes et al., 1999; Rozental et al., 2001; Dobrenis et al., 2005) but also through long intercellular processes termed tunneling nanotubes (TNTs) (Wang X. et al., 2012). These structures are F-actin-based cellular extensions that sustain direct interaction between neighboring cells and instead of filopodia and cytonemes, they permit the transfer of surface proteins and cytoplasmic content without touching the substrate (Abounit and Zurzolo, 2012) (Figure 1).

As already mentioned, hemichannels and pannexons constitute one of the pathways by which glial cells interact with neurons. During the past decade, a growing body of evidence begun to support a novel role for these channels as physiological modulators of synaptic efficacy, neural activity, signal processing, cognition and behavior (Huckstepp et al., 2010b; Stehberg et al., 2012; Torres et al., 2012; Chever et al., 2014; Retamal, 2014; Roux et al., 2015; Walrave et al., 2016; Meunier et al., 2017). The involvement of hemichannels and pannexons in higher brain functions relies on diverse mechanisms, yet the release of gliotransmitters seems to represent the most canonical and with potentially significant consequences for synaptic transmission. This article reviews and discusses recent evidence sustaining the “dual edge sword” role of hemichannels and pannexons in the function of central and peripheral synapses. According to this idea, in the healthy brain, these channels may act as pathways for the discharge of transmitters into the extracellular milieu to adjust the neural outcome in resting and stimulated conditions. In contrast, during pathological conditions, the persistent opening of hemichannels and pannexons could negatively impact the function and survival of brain cells.

## Hemichannel and Pannexon Opening as a Pathway Associated to the Release of Gliotransmitters

### General Characteristics of Hemichannels and Pannexons

**FIGURE 2:**
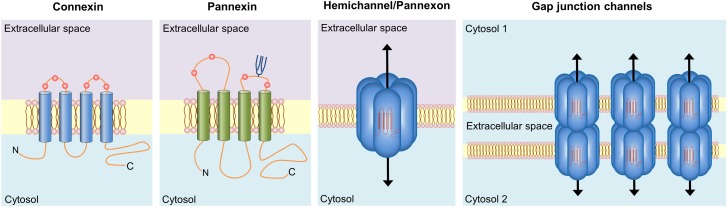
Basic structure of connexin and pannexin-based channels. Connexins and pannexins share a similar membrane topology with four α-helical transmembrane domains connected by two extracellular loops and one cytoplasmic loop; both the amino- and carboxy-termini are intracellular. The relative positions of the extracellular loop cysteines (red balls) and glycosylated asparagines (blue branches) are also shown. Hemichannels (also known as connexons) are formed by the oligomerization of six subunit connexins around a central pore. Pannexons are single membrane channels that are composed of six pannexin subunits. Recently, a band pattern more consistent with an octamer than a hexamer was observed in Panx2 by cross-linking studies and native gels of purified homomeric full-length and C-terminal truncation mutants (Ambrosi et al., 2010). Under resting conditions, hemichannels and pannexons remain preferentially closed, but they may be activated by diverse physiological and pathological conditions and offer a diffuse transmembrane route between the intra- and extracellular milieu. Hemichannels dock each other to form functional cell-to-cell channels termed gap junction channels (right panel). Gap junction channels aggregate in well-known anatomical structures called gap junctions to facilitate the intercellular cytoplasmic exchange of metabolites, second messengers and ions.

Connexins belong to a 21-member protein family that constitutes two distinct classes of plasma membrane channels: hemichannels and gap junction channels (GJCs). The former are constituted by the oligomerization of six connexin subunits around a central pore (Sáez et al., 2003). In spite of their low open probability (Contreras et al., 2003), when located in non-appositional plasma membranes and under certain physiological and pathological stimuli, hemichannels allow the movement of ions and small molecules -including ATP and glutamate- between the intracellular and extracellular space (Sáez et al., 2003) (Figure 2). On the other hand, GJCs derive from the serial docking of two hemichannels in appositional membranes that link the cytoplasm of two contacting cells. These channels permit the passive flow of ions and small molecules -such as cAMP, glucose and glutathione- between cells, ensuring metabolic and electrical coupling, as well as cellular coordination (Sáez et al., 2003) (Figure 2).

Pannexins are mammalian orthologs of innexins, the gap junction proteins of invertebrates. They are assembled in hexamers to form plasma membrane channels -known as pannexons- with similar topological and permeability properties than hemichannels (Ambrosi et al., 2010) (Figure 2). Three members encompass this family -Panx1, Panx2, and Panx3-, being Panx1 the most widely expressed in mammal tissues, including the brain and heart (Baranova et al., 2004). Just like hemichannels, pannexons serve as a route of ionic and molecular interchange between the cytoplasm and the extracellular compartment (Dahl, 2015). In spite of these similarities, pannexins and connexins do not share homologies in their sequence and nowadays whether pannexons are able to constitute GJCs *in vivo* is still matter of debate (Sosinsky et al., 2011). It is thought that *N*-glycosylation of pannexins at residues on the second extracellular loop (Penuela et al., 2014) may interfere with the ability of these channels to dock to each other and thus form gap junction-like structures.

Whereas hemichannels exhibit a low open probability at resting conditions (Contreras et al., 2003), pannexons open to more negative potentials than hemichannels and can be fully functional under resting membrane potentials (Bruzzone et al., 2003). Although both channels display larger membrane currents following increased depolarization, pannexons reach to maximum currents with faster kinetics and exhibit larger unitary conductance, weak voltage-gating, and several subconductance states when compared to hemichannels (Paul et al., 1991; White et al., 1999; Bruzzone et al., 2003; Bao et al., 2004). As opposed to most hemichannels, whose activity critically depend on the extracellular concentration of divalent cations (Ebihara and Steiner, 1993; Li et al., 1996; John et al., 1999; Contreras et al., 2003; Ebihara et al., 2003), gating properties of pannexons are not affected by external Ca^2+^ (Bruzzone et al., 2005). Indeed, at physiological Ca^2+^ concentrations pannexons seem quite functional (Bruzzone et al., 2005; Barbe et al., 2006) while most hemichannels show very low open probability (Ebihara and Steiner, 1993; Li et al., 1996; Pfahnl and Dahl, 1999; Valiunas, 2002; Ebihara et al., 2003). Consequently, lowering external Ca^2+^ is a commonly used maneuver to favor hemichannel opening (John et al., 1999). Despite the above evidence, recent reports have demonstrated that hemichannel opening during resting conditions is critical for basal synaptic transmission and long-term potentiation (Chever et al., 2014; Meunier et al., 2017).

Although the overlapping effects of some inhibitors have made pharmacology an insufficient criterion for distinguishing pannexons from hemichannels (Spray et al., 2006; Wang et al., 2007), they differ in their sensitivity to distinct blockers, including those commonly used to inhibit gap junctions (for an updated review, see Willebrords et al., 2017). For example, Panx1 channels are more sensitive than hemichannels to liquorice derivatives such as 18-α- and 18-β-glycyrrhetinic acid (α-, β-GA) and carbenoxolone (CBX), whereas flufenamic acid (FFA) and long-chain alcohols (e.g., octanol and heptanol) block GJC and hemichannel activity with minimal or no effect on pannexons (Harks et al., 2001; Eskandari et al., 2002; Braet et al., 2003; Bruzzone et al., 2005; Ma et al., 2009). Membrane-impermeant blockers La^3+^ and Gd^3+^ inhibit hemichannels with no impact on GJCs (John et al., 1999; Braet et al., 2003; Contreras et al., 2003) or Panx1 channels (Ma et al., 2009). Probenecid, an organic anion used for gout treatment, has proved to selectively counteract Panx1 channel opening in diverse preparations (Pelegrin and Surprenant, 2006; Silverman et al., 2008; Ma et al., 2009). Gap26 and Gap27, two mimetic peptides that interact with sequences of the extracellular loop regions of Cx43, inhibit GJCs and hemichannels at long (1–3 h) and short (1–30 min) periods of incubation, respectively (Chaytor et al., 1997; Evans and Boitano, 2001; Braet et al., 2003). Relevantly, the nonapeptide Gap19, derived from the cytoplasmic loop (CL) of Cx43, specifically blocks Cx43 hemichannels by preventing intramolecular C-terminus-CL interactions (Wang et al., 2013). On the other hand, the mimetic peptide ^10^Panx1, which interacts with an extracellular loop domain of Panx1, has been shown to prevent both current and molecular exchange mediated by Panx1 channels (Pelegrin and Surprenant, 2006; Thompson et al., 2006; Wang et al., 2007).

In healthy conditions, both hemichannels and pannexons have been implicated in physiological processes, such as visual processing in the retina (Klaassen et al., 2012; Cenedese et al., 2017), memory and learning (Prochnow et al., 2012; Stehberg et al., 2012), among other brain processes (reviewed in Cheung et al., 2014). Noteworthy, the impact of hemichannels in diverse biological processes seems in contradiction with their low open probability observed in exogenous expression systems (Contreras et al., 2003). However, most of these data were obtained at 21% O_2_, a very oxidant condition that does not match the arterial partial pressure of O_2_ (∼10–12%). The latter acquires particular relevance in the light of the increased opening probability that exhibit hemichannels in reducing conditions, specifically those formed by Cx43 (Retamal et al., 2007b). Thus, it is possible to speculate that *in vivo*, where O_2_ levels represent a less oxidant condition, hemichannel opening could be higher than expected from *in vitro* experiments. In contrast, the opening of pannexons in tissues could be lower than observed *in vitro*, as reducing agents decrease their activity (Bunse et al., 2009; Retamal, 2014).

At the other end, the open probability of hemichannels -*in vivo* and *in vitro-*, increases notably under pathological conditions. For instance, Cx43 hemichannels exhibit an augmented opening in astrocytes exposed to metabolic inhibition, inflammatory agents or redox imbalance (Contreras et al., 2002; Retamal et al., 2006, 2007a). Particularly, redox status may modulate connexin function in a complex way with different impacts on hemichannel opening. In fact, during healthy conditions reducing agents increase the activity of Cx43 hemichannels and similar responses are found upon treatment with oxidant molecules in certain pathological scenarios (Retamal et al., 2007b). How is it possible that reducing and oxidant agents can induce such equivalent effect? A conceivable explanation of this apparent contradiction is the intricated interplay between redox and phosphorylation status of Cx43 (Retamal et al., 2007b). Apparently, oxidant and reducing agents could oppositely influence or not hemichannel activity depending on the pattern of Cx43 phosphorylation (Retamal et al., 2007b). Another hypothesis is that modification of particular cysteine groups may induce contrasting impacts on hemichannel opening. Supporting this idea, cysteine modifications evoked by carbon monoxide (CO) and lipid peroxides induce Cx46 hemichannel closure (Leon-Paravic et al., 2014), whereas nitric oxide (NO)-mediated cysteine modulation of Cx46 leads to alterations in hemichannel-mediated currents and molecule permeation (Retamal et al., 2009).

Single point mutations in connexins may cause a high opening state of hemichannels, a phenomenon referred to as “leaky hemichannels” (Retamal et al., 2015). In this context, uncontrolled opening of hemichannels could lead to cell dysfunction and even cell death due to the loss of ion balance and membrane potential, as well as activation of detrimental cascades linked to Ca^2+^ overload (Li et al., 2001; Sanchez et al., 2010; Retamal et al., 2015). A coincident pattern has been documented for pannexons, as their opening underpins the genesis and progression of several diseases such as cancer (Lai et al., 2007, 2009; Penuela et al., 2012; Jiang and Penuela, 2016), epilepsy (Thompson et al., 2008; Kim and Kang, 2011; Santiago et al., 2011), overactive bladder (Timoteo et al., 2014), hypertension (Billaud et al., 2012), among other diseases (for more details, see Penuela et al., 2014). Unlike connexins, just one report has associated germline mutations affecting pannexin function with diseases. In this case, the mutation Arg217His of Panx1, induces intellectual disability, sensorineural hearing loss, kyphoscoliosis and primary ovarian failure (Shao et al., 2016).

### A Brief Description of Connexin and Pannexin Expression in Brain Cells

#### Neurons

Throughout the CNS, neurons display a wide expression of Cx36 and Cx45, both being major building blocks of the gap junction-based electrical synapse (Condorelli et al., 1998; Belluardo et al., 1999; Zhang and Restrepo, 2002; Connors and Long, 2004; Sohl et al., 2005; Belousov and Fontes, 2013; O’Brien, 2017) (Table 1). Other reports have shown that horizontal cells in the retina and neurons of the olfactory bulb also express Cx57 and Cx43, respectively (Zhang et al., 2000; Zhang, 2011; Pan et al., 2012), whereas the mRNAs for Cx37 and Cx40 have been detected in lumbar motor neurons (Chang et al., 1999) (Table 1). GJCs composed by Cx36 coordinate the synchronic and rhythmical firing of neurons organized in specific networks (Benardo and Foster, 1986; Meier and Dermietzel, 2006). Indeed, ablation of Cx36 impairs the synchronization but not the generation of oscillatory firing patterns in neural networks of the inferior olivary nucleus (Long et al., 2002). Also, small neurons of the thalamic reticular nucleus seem to be coupled and synchronized *via* Cx36 GJCs (Landisman et al., 2002; Zolnik and Connors, 2016). Supporting the role of Cx36 in higher brain function, its removal blunts the generation of widespread, synchronous inhibitory activity in the neocortex (Deans et al., 2001) and reduces gamma frequency (30–80 Hz) network oscillations without altering fast-field “ripple” (140–200 Hz) or theta (5–10 Hz) rhythms in the hippocampus (Hormuzdi et al., 2001; Buhl et al., 2003). In the retina, the electrical synapse between ON cone bipolar and AII amacrine cells relies on heterotypical GJCs composed by Cx36 and Cx45, respectively (Massey et al., 2003; Sohl et al., 2005) (Table 1). Deletion of Cx45 strongly disrupts the firing pattern of individual retinal ganglion cells during development (Blankenship et al., 2011). Relevantly, neuron-directed Cx45 deficient mice display impaired one-trial novel object recognition and kainate-mediated gamma-oscillations in the hippocampus (Zlomuzica et al., 2010).

**Table 1:** Brief summary of connexin and pannexin expression in the nervous system^∗^.

Cell type	Protein	Brain area	Experimental preparation	Specie	Evidence	Reference
Neuron	Cx36	Brainstem	Tissue and sections	Rat	ISH; NB; RT-PCR	Condorelli et al., 1998
		Cerebellum	Tissue and sections	Rat	ISH; NB; RT-PCR	Condorelli et al., 1998
			Tissue sections	Human	ISH	Belluardo et al., 1999
		Cerebral cortex	Tissue and sections	Rat	ISH; RT-PCR	Condorelli et al., 1998
			Tissue sections	Human	ISH	Belluardo et al., 1999
		Hippocampus	Tissue and sections	Rat	ISH; NB; RT-PCR	Condorelli et al., 1998
			Tissue sections	Human	ISH	Belluardo et al., 1999
		Hypothalamus	Tissue and sections	Rat	ISH; NB; RT-PCR	Condorelli et al., 1998
		Inferior olivary nuclei	Tissue and sections	Rat	ISH; NB; RT-PCR	Condorelli et al., 1998
			Tissue sections	Human	ISH	Belluardo et al., 1999
		Lumbar motor neurons	Tissue, sections and primary cultures	Rat	IF; ISH; RT-PCR	Chang et al., 1999
		Mesencephalon	Tissue and sections	Rat	ISH; NB; RT-PCR	Condorelli et al., 1998
			Tissue sections	Human	ISH	Belluardo et al., 1999
		Olfactory bulb	Tissue and sections	Rat	ISH; NB; RT-PCR	Condorelli et al., 1998
			Tissue sections	Human	ISH	Belluardo et al., 1999
		Pineal gland	Tissue and sections	Rat	ISH; NB; RT-PCR	Condorelli et al., 1998
		Reticular thalamic nucleus	Tissue and sections	Rat	ISH; RT-PCR	Condorelli et al., 1998
		Retina	Tissue	Rat	ISH; NB; RT-PCR	Condorelli et al., 1998
		Spinal cord	Tissue and sections	Rat	ISH; NB; RT-PCR	Condorelli et al., 1998
			Tissue sections	Human	ISH	Belluardo et al., 1999
		Striatum	Tissue and sections	Rat	ISH, RT-PCR	Condorelli et al., 1998
	Cx37	Lumbar motor neurons	Tissue, sections and primary cultures	Rat	IF; ISH; RT-PCR	Chang et al., 1999
	Cx40	Lumbar motor neurons	Tissue, sections and primary cultures	Rat	IF; ISH; RT-PCR	Chang et al., 1999
	Cx43	Lumbar motor neurons	Tissue, sections and primary cultures	Rat	IF; ISH; RT-PCR	Chang et al., 1999
		Olfactory bulb	Tissue and sections	Mouse	ISH; IF; WB;	Zhang et al., 2000
	Cx45	Lumbar motor neurons	Tissue, sections and primary cultures	Rat	IF; ISH; RT-PCR	Chang et al., 1999
		Olfactory bulb	Tissue and sections	Mouse	ISH; IF; WB	Zhang et al., 2000
	Cx57	Olfactory bulb	Tissue and sections	Mouse	IF; ISH; qPCR; RT-PCR	Zhang, 2011
		Retina	Tissue and sections	Rabbit	IF; RT-PCR	Pan et al., 2012
	Panx1	Cerebellum	Tissue sections	Rat	IHC; ISH	Vogt et al., 2005
			Tissue sections	Rat	ISH	Bruzzone et al., 2003
		Cerebral cortex	Tissue sections	Rat	IHC; ISH	Vogt et al., 2005
			Tissue sections	Rat	ISH	Bruzzone et al., 2003
		Diencephalon	Tissue sections	Rat	IHC; ISH	Vogt et al., 2005
		Hippocampus	Tissue sections	Rat	IHC; ISH	Vogt et al., 2005
			Tissue sections	Rat	ISH	Bruzzone et al., 2003
			Tissue sections, primary cultures	Mouse, rat	EM; IF; IHC;	Zoidl et al., 2007
		Olfactory bulb	Tissue sections	Rat	IHC; ISH	Vogt et al., 2005
			Tissue sections	Rat	ISH	Bruzzone et al., 2003
		Retina	Tissue and sections	Mouse, rat	IF; ISH; qPCR; WB	Dvoriantchikova et al., 2006
	Panx2	Cerebellum	Tissue sections	Rat	IHC; ISH	Vogt et al., 2005
			Tissue sections	Rat	ISH	Bruzzone et al., 2003
		Cerebral cortex	Tissue sections	Rat	IHC; ISH	Vogt et al., 2005
			Tissue sections	Rat	ISH	Bruzzone et al., 2003
		Hippocampus	Tissue sections	Rat	IHC; ISH	Vogt et al., 2005
			Tissue sections	Rat	ISH	Bruzzone et al., 2003
		Olfactory bulb	Tissue sections	Rat	IHC; ISH	Vogt et al., 2005
			Tissue sections	Rat	ISH	Bruzzone et al., 2003
		Retina	Tissue and sections	Mouse, rat	ISH; qPCR;	Dvoriantchikova et al., 2006
		Thalamus	Tissue sections	Rat	ISH; IHC	Vogt et al., 2005
			Tissue sections	Rat	ISH	Bruzzone et al., 2003
Astrocyte	Cx26	Paraventricular nucleus	Tissue sections	Rat	FRIL; IF	Nagy et al., 2001
		Spinal cord	Tissue sections	Rat	FRIL; IF	Nagy et al., 2001
	Cx30	Cortex	Tissue, sections, and primary cultures	Rat	IF; WB	Kunzelmann et al., 1999
		Hippocampus	Tissue sections	Mouse	IF	Kunzelmann et al., 1999
			Tissue sections	Mouse	FA; IF; NB; TG; WB	Gosejacob et al., 2011
		Reticular thalamic nucleus	Tissue sections	Rat	EM	Nagy et al., 1999
		Subthalamic nucleus	Tissue sections	Rat	EM	Nagy et al., 1999
	Cx43	Cerebral cortex	Tissue sections	Rat	EM	Yamamoto et al., 1990
			Primary cultures	Rat	IF; IHC; NB; WB	Dermietzel et al., 1991
		Corpus callosum	Tissue sections	Rat	EM	Yamamoto et al., 1990
		Dorsal tegmental brainstem	Tissue sections	Rat	EM	Yamamoto et al., 1990
		Striatum	Primary cultures	Rat	IF; IHC; NB; WB	Dermietzel et al., 1991
			Primary cultures	Mouse, rat	IF; IHC; NB; WB	Giaume et al., 1991
Oligodendrocytes	Cx29	Cerebellum	Tissue sections	Mouse	NB	Altevogt et al., 2002
		Cerebrum	Tissue sections	Mouse	NB	Altevogt et al., 2002
		Spinal cord	Tissue sections	Mouse	IF; ISH; NB	Altevogt et al., 2002
	Cx31.1	Cerebral cortex	Tissue sections	Human	IF	Sargiannidou et al., 2008
	Cx32	Basal ganglia	Tissue sections	Rat	IF	Dermietzel et al., 1989
		Brain stem	Tissue sections	Rat	IF	Dermietzel et al., 1989
			Tissue sections	Rat	IF	Dermietzel et al., 1997
		Cerebral cortex	Tissue sections	Rat	IF	Dermietzel et al., 1989
			Tissue sections	Human	IF	Sargiannidou et al., 2008
		Cerebrum	Primary cultures	Bovine	IF; NB; SB; WB	Dermietzel et al., 1997
		Hippocampus	Tissue sections	Rat	IF	Kunzelmann et al., 1997
		Spinal cord	Tissue sections	Mouse	IF	Altevogt et al., 2002
			Tissue sections	Rat	IF	Dermietzel et al., 1997
		Thalamus	Tissue sections	Rat	IF	Dermietzel et al., 1989
	Cx45	Brain stem	Tissue sections	Rat	IF	Dermietzel et al., 1997
		Cerebrum	Primary cultures	Bovine	IF; NB; SB; WB	Dermietzel et al., 1997
		Hippocampus	Tissue sections	Rat	IF	Dermietzel et al., 1997
			Tissue sections	Rat	IF	Kunzelmann et al., 1997
		Spinal cord	Tissue sections	Rat	IF	Dermietzel et al., 1997
	Cx47	Brain stem	Tissue sections	Mouse	IF	Li et al., 2004
		Cerebellum	Tissue sections	Mouse	IF	Odermatt et al., 2003
			Tissue sections	Mouse	IF	Li et al., 2004
		Cortex	Tissue sections	Mouse	IF	Li et al., 2004
		Corpus callosum	Tissue sections	Mouse	IF	Odermatt et al., 2003
		Hippocampus	Tissue sections	Mouse	IF	Li et al., 2004
		Hypothalamus	Tissue sections	Mouse	IF	Li et al., 2004
		Spinal cord	Tissue sections	Rat	FRIL	Li et al., 2004
		Thalamus	Tissue sections	Mouse	IF	Li et al., 2004
Microglia	Cx29	Cortex	Tissue sections	Mouse	IF	Moon et al., 2010
	Cx32	Cerebrum	Primary cultures	Mouse	FC	Takeuchi et al., 2006
			Primary cultures	Mouse	IF; RT-CR; WB	Maezawa and Jin, 2010
		Cortex	Tissue sections	Mouse	IF	Moon et al., 2010
	Cx36	Cerebrum	Primary cultures	Rat	IHC; RT-PCR	Parenti et al., 2002
		Neocortex	Primary cultures	Human	RT-PCR; WB	Dobrenis et al., 2005
			Primary cultures	Mouse	RT-PCR; WB	Dobrenis et al., 2005
	Cx43	Cortex	Tissue sections	Rat	IF	Eugenin et al., 2001
			Primary cultures	Rat	IF; WB	Eugenin et al., 2001
			Primary cultures	Rat	IF; RT-PCR; WB	Martinez et al., 2002
			Primary cultures	Rat	IF; WB	Sáez et al., 2013b
	Cx45	Neocortex	Primary cultures	Mouse	RT-PCR; WB	Dobrenis et al., 2005
	Panx1	Cortex	Primary cultures	Rat	IF; WB	Sáez et al., 2013b

*This table was intented to show several examples and does not correspond to a compilation of all published evidence. EM, electron microscopy; IF, immunofluorescence; IHC, immunohistochemistry; ISH, in situ hybridization; FACS, fluorescence activated cell sorter; FC; flow cytochemistry; FRIL, freeze-fracture replica immunogold labeling; WB, Western blot; NB, Northern blot; SB, Southern blot; RT-PCR, reverse transcriptase polymerase chain reaction; qPCR, real time or quantitative polymerase chain reaction.*

Both Panx1 and Panx2 are broadly expressed at the CNS (Bruzzone et al., 2003; Vogt et al., 2005; Dvoriantchikova et al., 2006; Zoidl et al., 2007) (see Table 1). Pioneering findings by MacVicar’s Lab showed that oxygen and glucose deprivation triggers single-large conductance channels formed by Panx1 in hippocampal neurons (Thompson et al., 2006). Follow-up studies found that stimulation of *N*-methyl-D-aspartate receptors (NMDARs) in pyramidal neurons activates Panx1 channel opening via Src family kinases, contributing to epileptiform seizure activity and excitotoxicity (Thompson et al., 2008; Weilinger et al., 2012).

#### Astrocytes

Under physiological conditions, rat, mouse and human astrocytes express abundantly Cx30 and Cx43 (Yamamoto et al., 1990; Dermietzel et al., 1991; Kunzelmann et al., 1999; Nagy et al., 1999; Giaume et al., 2010), whereas some evidence indicates that they also can express Cx26 (Nagy et al., 2001) (Table 1). However, their relative expression shows a heterogeneous pattern in astrocytes and changes depending on the developmental stage and brain region (Batter et al., 1992; Zhang et al., 1999; Nagy and Rash, 2000; Gosejacob et al., 2011) (Table 1). Of note, Cx43 ablation reduces hippocampal astrocyte coupling by 50%, whereas deletion of both Cx30 and Cx43, completely abolishes astrocyte-astrocyte coupling (Gosejacob et al., 2011). In the same line, Cx30-deficient mice display anxiogenic behavior and reduced rearing activity correlated with increased choline levels in the ventral striatum (Dere et al., 2003). Relevantly, Cx43-mediated gap junction coupling underpins the spreading of intracellular K^+^, Na^+^, and Ca^2+^ (Cotrina et al., 1998; Scemes et al., 1998; Wallraff et al., 2006; Langer et al., 2012), participating thus in K^+^ buffering, maintenance of neuronal membrane potential and coordination of large populations of astrocytes, all processes being critical for synaptic transmission (Pannasch et al., 2011, 2014; Chever et al., 2014, 2016). Additionally, Cx43 GJCs mediate glucose and lactate trafficking among astrocytes (Ball et al., 2007; Rouach et al., 2008). Moreover, astrocytes actively provide glucose to neurons when they needed and remove lactate from high activity areas (Gandhi et al., 2009). When this gap junction-dependent “energy” flux is impaired, the sleep-wake cycle is disturbed as a result of a decrease in orexinergic neurons in the lateral hypothalamus (Clasadonte et al., 2017). Notably, the excessive sleepiness is prevented by the application of lactate to this brain area. The latter suggests that metabolic coordination between astrocytes and neurons is fundamental for certain brain functions. Astroglial Cx43 hemichannels have been observed *in vitro* and *ex vivo* (Karpuk et al., 2011; Chever et al., 2014; Abudara et al., 2015) and their opening seems to underlie the release of gliotransmitters -such as ATP (Stout et al., 2002) and glutamate- (Ye et al., 2003), with potentially relevant consequences for higher brain function *in vivo* (Stehberg et al., 2012; Vazquez et al., 2015; Walrave et al., 2016).

#### Oligodendrocytes

Oligodendrocytes are the myelin-producing cells at the CNS and express several types of connexins, including Cx29 in mice or its human orthologous Cx31.1 (Altevogt et al., 2002; Sargiannidou et al., 2008), Cx32 (Dermietzel et al., 1989), Cx45 (Dermietzel et al., 1997; Kunzelmann et al., 1997) and Cx47 (Odermatt et al., 2003; Li et al., 2004) (Table 1). Among them, Cx32 has been the most studied, probably because its mutation causes progressive loss of myelin and muscle weakness along with other complex manifestations that together are known as the X-linked Charcot-Marie-Tooth disease (Ressot et al., 1998; Yoshimura et al., 1998; Kleopa et al., 2012; Wang and Yin, 2016). Freeze-fracture microscopy has revealed that oligodendrocytes form heterotypical GJCs with astrocytes (Rash et al., 1998), with Cx43 and Cx45 being the putative contributors from the astroglial and oligodendrocyte side, respectively (Nagy and Rash, 2000). Nevertheless, confocal studies and electron microscopy suggest that oligodendrocyte-to-astrocyte coupling may proceed through Cx43/Cx47, Cx30/Cx32, and Cx26/Cx32 GJCs (Altevogt and Paul, 2004; Wasseff and Scherer, 2011; Tress et al., 2012). Although several hypotheses have been proposed to explain the role of astrocyte-to-oligodendrocyte coupling (Orthmann-Murphy et al., 2008), recent evidence demonstrates its importance for accurate myelin function and homeostasis of the CNS (Tress et al., 2012; May et al., 2013), as well as glucose spreading (Niu et al., 2016). The latter study provided the unique evidence of the physiological role of hemichannels in oligodendrocytes and oligodendrocyte precursor cells (OPCs). They found that hemichannels allow the influx of glucose in oligodendrocytes and OPCs along with contributing to OPC proliferation by a mechanism involving the elevation of intracellular free Ca^2+^ concentration ([Ca^2+^]_i_) (Niu et al., 2016). Panx1 channels are also expressed by oligodendrocytes where in association with P2X_7_ receptors they mediate ischemic damage (Domercq et al., 2010).

#### Microglia

In resting conditions, both Cx32 and Cx36 have been detected in microglia by immunofluorescence and RT-PCR (Parenti et al., 2002; Maezawa and Jin, 2010) (Table 1). Cx36 *in vitro* has been proposed to underpin gap junctional communication between microglia and neurons, although the biological relevance is uncertain as barely 30% and 4% of electrophysiological and dye diffusion experiments resulted in successful coupling, respectively (Dobrenis et al., 2005). Once activated during different pathological conditions, microglia display increased levels of Cx29 (Moon et al., 2010), Cx32 (Maezawa and Jin, 2010) and Cx43, the latter likely underlying the formation of functional GJCs (Eugenin et al., 2001; Martinez et al., 2002). Despite the biological significance of microglial coupling remains elusive, it has been hypothesized that gap junctions are crucial for ruling dynamic changes in microglial phenotype, the exchange of antigen peptides between activated microglia or the cross-presentation of antigens to T cells (Gajardo-Gomez et al., 2017). Pioneering studies by Takeuchi et al. (2006) showed that TNF-α-mediated upregulation of Cx32 hemichannels contributes to the exacerbated release of glutamate and subsequent neuronal beading and death. From there on, different inflammatory agents -including Aβ, LPS and ATP have been described to increase the opening of hemichannels formed by Cx43 and Cx32, as well as Panx1 channels (Sáez et al., 2013b), the latter being of substantial impact for gliotransmission and excitotoxicity (Gajardo-Gomez et al., 2017).

### The Release of Gliotransmitters Through Hemichannels and Pannexons

#### ATP Release

At the end of the 1990s, Cotrina et al. (1998) demonstrated that C6-glioma cells transfected with Cx32 or Cx43 show a prominent ATP release compared with mock C6 cells. A few years later, Stout and co-workers using cultures of mouse astrocytes and C6-Cx43 glioma cells, measured the presence of active hemichannels through whole-cell patch clamp and dye uptake experiments (Stout et al., 2002). Additionally, in both astrocytes and C6-Cx43 cells, mechanical stimulation caused a strong release of ATP, detected as an increase of luciferin-luciferase bioluminescence. This response was either blocked by 50 μM Gd^3+^ or 50 μM FFA and potentiated by a Ca^2+^-free solution (a well-established condition that opens hemichannels). Because ATP release was enhanced with zero extracellular Ca^2+^ and blunted by classic -but unspecific- hemichannel blockers, Cx43 hemichannels were suggested as possible mediators of this response (Stout et al., 2002). Later, elegant experiments by Nedergaard’s Laboratory demonstrated that glial Cx43 hemichannels are permeable to ATP, as measured by simultaneous single-channel recordings and bioluminescence imaging (Kang et al., 2008). Other studies have found a similar pattern of ATP release in astrocytes during either physiological or pathological conditions (Orellana et al., 2011a,b; Huang et al., 2012; Chever et al., 2014). The release of ATP takes place also through Panx1 channels in astrocytes (Iglesias et al., 2009; Suadicani et al., 2012; Garre et al., 2016) and microglia (Higashi et al., 2011; Orellana et al., 2013), whereas in tanycytes it depends on Cx43 hemichannels and Panx1 channels as well (Orellana et al., 2012; Lazutkaite et al., 2017).

#### Glutamate Release

Ye et al. (2003) provided the first evidence that hemichannels are implicated in the release of glutamate in primary cultured astrocytes. They observed that removing extracellular Ca^2+^ and Mg^2+^, increased the efflux of glutamate, taurine and aspartate, these responses being dramatically suppressed by different general hemichannel blockers (e.g., CBX, octanol, heptanol and La^3+^). Hemichannel-dependent release of glutamate is enhanced by exposing astrocytes to hypertonic solutions (Jiang et al., 2011), infrasound (16 Hz, 130 dB) (Jiang et al., 2014), Aβ (Orellana et al., 2011b) or LPS (Abudara et al., 2015). Astrocytes are not the only non-neuronal cells that can release glutamate through hemichannels. As already mentioned, TNF-α induces Cx32 hemichannel opening in microglia and the subsequent release of glutamate through them, effect that was sensitive to mimetic peptides against Cx32 (Takeuchi et al., 2006). On the other hand, rat retinal glial (Müller) cells (Voigt et al., 2015) and satellite glial cells (Wagner et al., 2014) also release glutamate in a hemichannel dependent form. In addition to hemichannels, pannexons formed by Panx1 may also contribute to the release of glutamate from glial cells. Thus, U87 cells derived from malignant glioma release important amounts of glutamate, the latter being dramatically decreased upon transfection with siRNA against Panx1 (Wei et al., 2016). Similarly, Panx1 channels contribute to the glutamate release from cerebrocortical synaptosomes (Di Cesare Mannelli et al., 2015) and astrocytes (Wei et al., 2014) evoked by oxaliplatin and ultrafine carbon black particles, respectively.

#### D-Serine Release

Despite the lack of direct evidence of D-serine being released through hemichannels or pannexons, a couple of works have strongly suggested this possibility. TAT-L2, a specific mimetic peptide against Cx43 hemichannels, greatly reduces fear memory consolidation when microinjected in the basolateral amygdala (BLA) (Stehberg et al., 2012). Noteworthy, the TAT-L2-mediated amnesic effects were rescued by a mixture of gliotransmitters microinjected at the BLA, including D-serine, supporting that Cx43 hemichannels could be implicated in its release. Similarly, evidence from Giaume’s Laboratory has suggested that NMDA-dependent synaptic transmission in the prefrontal cortex may need the release of D-serine via the aperture of astroglial Cx43 hemichannels (Meunier et al., 2017). The efflux of astroglial D-serine has also been related to the opening of Panx1 channels (Pan et al., 2015).

## Connexons and Pannexons at the Tripartite Synapse: a Feedback Mechanism to Reset the Strength of Neurotransmission

**FIGURE 3:**
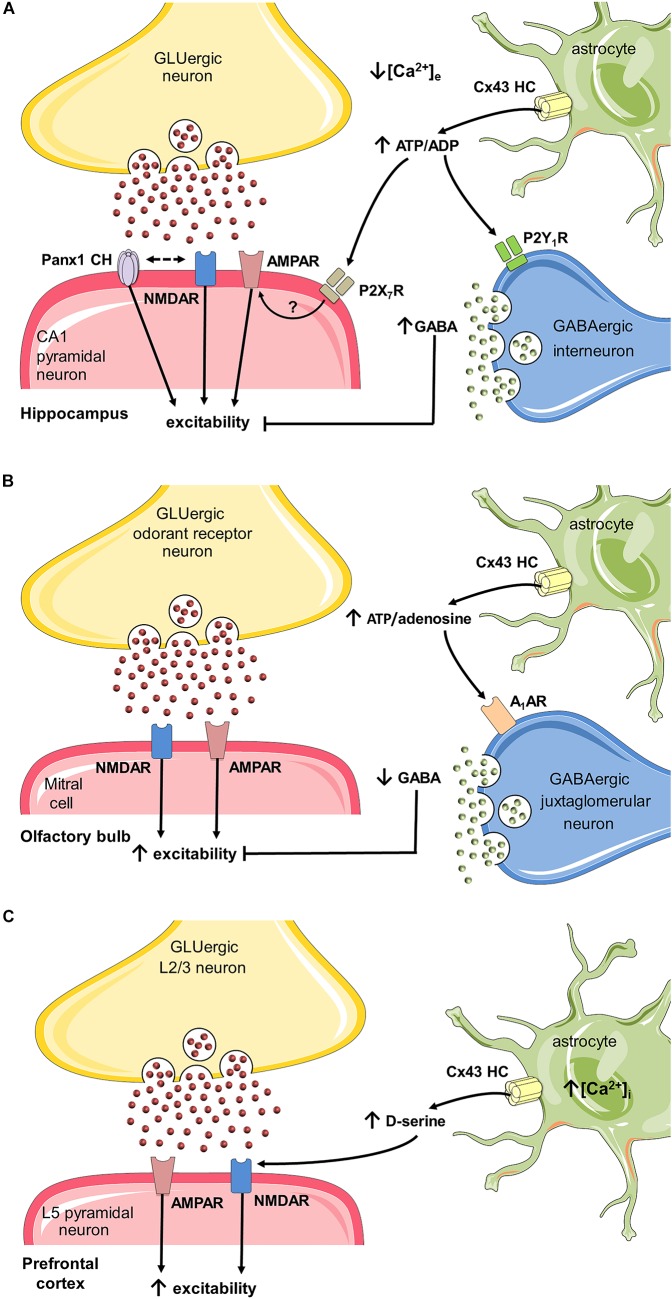
Possible roles of hemichannels and pannexons in synaptic plasticity through activation of astrocytes. **(A)** During basal glutamatergic signaling in the hippocampus, Ca^2+^ influx into neurons leads to a localized reduction in [Ca^2+^]_e_, which in turn opens Cx43 hemichannels (HCs) on astrocytes (Torres et al., 2012), resulting in the release of ATP. In the synaptic cleft, this gliotransmitter sustains basal excitatory synaptic transmission (Chever et al., 2014), (which may depend on the activation of P2X_7_ receptors and further insertion of AMPA receptors in postsynaptic terminals (Gordon et al., 2005). The interacting coupling between NMDARs and Panx1 channels could be a possible mechanism to potentiate the above response (Weilinger et al., 2012), though pannexons may also restrain synaptic plasticity (Ardiles et al., 2014) (not depicted). Alternatively, the conversion of ATP to ADP could depolarize and increase firing in interneurons via P2Y_1_ receptors, therefore, enhancing inhibitory transmission (Torres et al., 2012). **(B)** Spontaneous neuronal activity in the olfactory bulb glomerular layer requires glutamatergic transmission. Under these conditions, astrocytes exhibit a basal function of Cx43 HCs that underpins the release of ATP (Roux et al., 2015). The adenosine derived from ATP may suppress the activity of GABAergic inhibitory juxtaglomerular neurons via the stimulation of A_1_ adenosine receptors. The latter allows the basal slow oscillations of up and down states of mitral cells in the olfactory bulb. **(C)** In the prefrontal cortex, sustained stimulation of layer 2/3 neurons produces long term potentiation (LTP) of NMDA and AMPA receptor currents in layer 5 pyramidal neurons. In this scenario, [Ca^2+^]_i_ is necessary for the opening of Cx43 HCs in astrocytes (Meunier et al., 2017), which leads to the release of D-serine. This gliotransmitter favors LTP of NMDA and AMPA excitatory synaptic currents triggered by high-frequency stimulation in the prefrontal cortex.)

Whereas gliotransmission at the tripartite synapse mostly relies on intracellular Ca^2+^-dependent exocytotic release, astroglial hemichannels and pannexons arise as alternative non-vesicular routes for gliotransmitter efflux to either attenuate or potentiate neurotransmission (Parpura et al., 2004; Huckstepp et al., 2010b; Torres et al., 2012; Montero and Orellana, 2015; Meunier et al., 2017). Direct evidence implicating astrocyte hemichannels as both sensors and modulators of synaptic activity comes from original studies in hippocampal slices by Torres et al. (2012). They found that UV-photolysis of caged MNI-glutamate, which depolarizes neurons by increasing extracellular glutamate reduces local extracellular Ca^2+^ concentration ([Ca^2+^]_e_) and enhances the release of glial ATP with the consequent spread of fast and slow Ca^2+^ waves in astrocytes (Torres et al., 2012). Relevantly, the specific deletion of Cx30/Cx43, but not Cx30 in astrocytes, eliminated the ATP-dependent spreading of slow Ca^2+^ waves triggered by photolysis of caged MNI-glutamate. Supporting these data, slices from transgenic mice with an astrocyte-targeted point mutation (Cx43G138R) that leads to an increased Cx43 hemichannel opening (Dobrowolski et al., 2008), potentiated the spreading of slow Ca^2+^ waves evoked by photolysis of MNI-glutamate (Torres et al., 2012). In addition, the authors observed that during low [Ca^2+^]_e_ (induced by either increasing extracellular glutamate or through high-frequency stimulation) depolarization of inhibitory interneurons from the stratum radiatum blunts CA1 excitatory transmission *via* the Cx43-hemichannel mediated release of astroglial ATP and further activation of interneuronal P2Y_1_ receptors. Therefore, the authors proposed that astrocyte Cx43 hemichannels provide a negative feedback mechanism elicited during sustained excitation to prevent excitotoxicity (Torres et al., 2012) (Figure 3A). Although the physiological significance of lowering [Ca^2+^]_e_ to levels reached in this study has been questioned (Cheung et al., 2014) and its extrapolation toward *in vivo* requires further investigation, this report is pioneering in unveiling how hemichannel-mediated gliotransmission participates at the central tripartite synapse.

ATP has emerged as a primary candidate released through astroglial hemichannels and pannexons to influence neural functions. Accordingly, ATP modulates neuro-glial interactions (Newman, 2003; Volterra and Meldolesi, 2005); and its permeation through Cx43 hemichannels has already been demonstrated (Kang et al., 2008). Albeit under resting potential and normal extracellular levels of Ca^2+^/Mg^2+^, Cx43 hemichannels exhibit a low open probability *in vitro* (Contreras et al., 2003), recent findings show that they may allow the release of ATP in acute brain slices under basal conditions (Chever et al., 2014; Roux et al., 2015). In this respect, the basal release of ATP via astroglial Cx43 hemichannels at the Stratum radiatum, is sufficient to enhance the CA1 synaptic transmission elicited by stimulation of Schaffer collaterals, an effect mediated by purinergic receptors (Chever et al., 2014) (Figure 3A). Although the machinery by which ATP released from astrocytes potentiates glutamatergic synaptic transmission is still unknown, the insertion of postsynaptic AMPA receptors as result of the activation of P2X_7_ receptors could be a possibility, as previously demonstrated in other brain regions (Gordon et al., 2005). The finding that astrocyte hemichannels boost glutamatergic synaptic transmission in resting conditions, brings down the common belief of hemichannels as iconographic pathways contributing to the cellular damage. In the same line, recently, astroglial Cx43 hemichannels were found essential for modulating neuronal network oscillations in the olfactory bulb (OB) (Roux et al., 2015). Normally, whole-cell current recordings of mitral cells at the OB acute slices display spontaneous alternations between depolarized (UP) states linked with spikes and silent hyperpolarized (DOWN) states (Carlson et al., 2000; Schoppa and Westbrook, 2001). Whereas the frequency and duration of these oscillations were independent of hemichannel activity, mitral cells of OB slices with specific astroglial deletion of both Cx30 and Cx43 showed a decreased firing and amplitude of UP states (Roux et al., 2015) (Figure 3B). These changes were also observed when Cx43 but not Cx30 was specifically deleted in astrocytes or after pharmacological inhibition of Cx43 hemichannels with Gap26 suggesting the involvement of Cx43 rather than Cx30 hemichannels. Gap26 is a mimetic peptide against the first extracellular loop of Cx43 that blocks hemichannels within minutes (Wang N. et al., 2012), but also the gap junction coupling at longer periods (>2–3 h), as it impedes the docking of hemichannels at appositional membranes (Evans and Leybaert, 2007). As demonstrated in other brain areas (Kang et al., 2008; Torres et al., 2012; Chever et al., 2014), bioluminescence assays, as well as pharmacological and genetic evidence revealed that Cx43 hemichannels contribute to the release of ATP in the OB (Roux et al., 2015). In this brain region, the ecto-5′-nucleotidase that catalyzes the conversion of adenosine from ATP is highly expressed, favoring the ATP/adenosine balance to adenosine (Langer et al., 2008). Therefore, the authors hypothesized that adenosine originated as the breakdown of ATP released via Cx43 hemichannels may modulate the firing pattern of mitral cells (Figure 3B). To test this, they applied adenosine receptor blockers in bath solution of OB slices. Only inhibition of A1 receptors reduced the amplitude of UP states and the firing rate of mitral cells, revealing that astrocyte Cx43 hemichannels increase the amplitude of UP states of mitral cells through the release of ATP and its further breakdown to adenosine (Roux et al., 2015). Because the usual mediated effects of A1 receptors include presynaptic inhibition of glutamate release, reduced postsynaptic NMDAR activation and decreased Ca^2+^ influx (Benarroch, 2008), possibly the enhancement of UP states by adenosine likely rely on A1-receptor mediated suppression of inhibitory juxtaglomerular interneurons (Figure 3B), as occurs in other brain areas (Morairty et al., 2004).

Recent evidence indicates that astroglial Cx43 hemichannels potentiate synaptic transmission at the prefrontal cortex (PFC) through the release of D-serine (Meunier et al., 2017). It has been shown that D-serine is a co-agonist of NMDARs, the main player underlying central excitatory glutamatergic transmission and synaptic plasticity (Panatier et al., 2006). Both astrocytes and neurons are now accepted as brain sources of D-serine (Martineau et al., 2014). In fact, a mounting body of data suggests that hippocampal astrocytes control long-term potentiation (LTP) by releasing D-serine (Yang et al., 2003; Henneberger et al., 2010). Indeed, previous reports have described that efflux of astroglial D-serine can take place through both Ca^2+^-dependent exocytosis (Mothet et al., 2005) or the opening of Panx1 channels by Ca^2+^-independent activation of P2X_7_ receptors (Pan et al., 2015). At the PFC, where D-serine and serine racemase exhibit high levels (Hashimoto et al., 1995; Fossat et al., 2012), the [Ca^2+^]_i_-dependent activation of astrocyte Cx43 hemichannels leads to D-serine efflux and subsequent enhancing of LTP (Meunier et al., 2017). In acute PFC slices, neuronal stimulation of layer 2/3 (L2/3) causes glutamatergic synaptic transmission in pyramidal cells at the layer 5 (L5) (DeNardo et al., 2015). In this context and using PFC slices of young mice, Meunier et al. (2017) found that Gap26 prominently blunts the NMDAR-dependent excitatory postsynaptic currents (EPSCs) and increases AMPA/NMDA current ratio in L5, an effect strongly prevented by the exogenous addition of D-serine (Figure 3C). Since 2-week-old mice express Cx43 uniquely in astrocytes (Nagy and Rash, 2000), short-term (minutes) application of Gap26 into slices was assumed to only target astrocyte Cx43 hemichannels. Furthermore, genetic ablation of Cx43 in astrocytes evoked a similar reduction in EPSCs and elevation of AMPA/NMDA current ratio in L5 of the PFC. Altogether, these results suggest that release of D-serine and astroglial hemichannel function are associated and regulate NMDAR-dependent synaptic transmission in PFC pyramidal cells (Figure 3C). As evidenced by dye uptake experiments, Meunier et al. (2017) showed that increasing [Ca^2+^]_i_ in cultured astrocytes opens Cx43 hemichannels. With this in mind, they further examined whether this mechanism may contribute to LTP in PFC slices. For this purpose, [Ca^2+^]_i_ was clamped in the L5 astroglial network while recording NMDAR-dependent EPSCs in neighboring L5 pyramidal cells in response to HFS protocol in L2/3. When [Ca^2+^]_i_ was clamped in the L5 astroglial network, HFS failed to potentiate the NMDAR-dependent synaptic currents, the latter response being also detected upon addition of the Cx43 hemichannel blocker Gap26 (Meunier et al., 2017). Finally, to address the involvement of astroglial D-serine in the above responses, its synthesis was blocked by delivering the serine racemase inhibitor L-erytho-3-hydroxyaspartate (HOAsp) specifically in astrocytes with a patch pipette. HOAsp has a low molecular weight (148 Da) which enables its diffusion within the GJC-mediated astroglial network. Importantly, HOAsp infusion prevented the HFS-induced potentiation of NMDAR-dependent currents; a phenomenon partially rescued by adding extracellular D-serine (Meunier et al., 2017). Altogether, these results imply that potentiation of glutamatergic transmission at the PFC depends on [Ca^2+^]_i_-mediated opening of astroglial Cx43 hemichannels and the consequent release of D-serine (Figure 3C).

The involvement of astroglial hemichannels in synaptic transmission has been correlated with their impact on higher brain function and behavior. As already mentioned in this article, *in vivo* blockade of Cx43 hemichannels at the BLA induces transitory and specific amnesia for auditory fear conditioning (Stehberg et al., 2012). Notably, learning capacity was recuperated by the co-administration of a cocktail of presumed gliotransmitters (lactate, glutamate, D-serine, glutamine, glycine and ATP), evidencing for the first time a physiological participation for astroglial Cx43 hemichannels in higher brain function. Recently, using a similar approach, these channels were reported to contribute to spatial short-term memory (Walrave et al., 2016). Intraventricular administration of the mimetic peptide Gap19 -which specifically blocks Cx43 hemichannels but not GJCs (Wang et al., 2013)- was found to significantly impair the spatial short-term memory, as examined with the delayed spontaneous alternation Y maze task (Walrave et al., 2016).

Panx1 channels have raised as crucial protagonists in the modulation of synaptic transmission and higher brain functions. In fact, total deletion of Panx1 enhances the amplitude of field excitatory postsynaptic potentials (fEPSPs) at hippocampal Schaffer collateral-CA1 synapses, an effect partially prevented by the exogenous application of adenosine (Prochnow et al., 2012). Furthermore, Panx1^-/-^ mice exhibit increased anxiety and disturbed object recognition and spatial learning (Prochnow et al., 2012). It is known that adult Panx1^-/-^ mice display both a long-lasting depletion of extracellular ATP in brain slices and cultured astrocytes (Santiago et al., 2011; Suadicani et al., 2012) and a compensatory up-regulation of metabotropic glutamate type 4 receptors (mGluR4s) (Prochnow et al., 2012). In consequence, the authors proposed that Panx1 channel-mediated release of ATP provides a feedback mechanism for counteracting hippocampal excitatory transmission, in where presynaptic activation of adenosine A1 receptors and the resulting inhibition of glutamatergic release adjust the synaptic strength within an effective range (Prochnow et al., 2012).

## Hemichannels and Physiological Function: Evidence From the Central and Peripheral Chemoreflex Control of the Ventilation

The homeostatic ventilatory response during chronic or acute exposure to high CO_2_/pH depends on the activity of central chemoreceptors. Several chemosensitive areas within the brainstem have been identified as crucial players in governing the central chemoreflex drive, such as the retrotrapezoid nucleus (RTN), parafacial respiratory group, raphe nuclei, the Pre-Bötzinger complex and ventral medullary surface (VMS) of the medulla oblongata (Nattie and Li, 2012; Guyenet, 2014). Particularly, a study in the VMS brought up the first compelling evidence linking the function of hemichannels with central respiratory CO_2_ chemosensitivity. Analyzing the VMS in *ex vivo* slices, Huckstepp et al. (2010b) found that CO_2_-dependent release of ATP, a major transmitter involved in hypercapnic ventilatory response (Gourine et al., 2005a,b), was insensitive to the Panx1 channel blocker probenecid, but sensitive to concentrations in which CBX act as hemichannel and GJC inhibitor (Huckstepp et al., 2010b). Because CO_2_-mediated ATP release at the VMS took place along with dye uptake in subpial and perivascular astrocytes expressing Cx26, hemichannels composed by this connexin were proposed as major contributors to this response. This assumption found plausibleness at the light of in vitro data in HeLa cells, where Cx26 transfection was enough to give them the capacity to release ATP and display hemichannel currents upon CO_2_ treatment (Huckstepp et al., 2010a). Later evidence revealed that a carbamate bridge between Lys125 and Arg104 might serve as a CO_2_ sensor in Cx26 (Meigh et al., 2013).

Follow-up studies uncovered that hemichannels also have a chemoreceptive role in the RTN. This nucleus is one of the main central chemoreceptor regions since it accounts for almost 90% of the total ventilatory response during hypercapnic stimulation (Takakura et al., 2014; Kumar et al., 2015). The precise mechanisms that confer CO_2_/pH sensitivity to RTN neurons seem to rely on the expression of both the pH-sensitive G-coupled receptor 4 (GPR4) and the background K^+^ channel (TASK-2), the latter reducing its activity in response to acidosis (Gestreau et al., 2010; Kumar et al., 2015). A growing body of evidence suggests that ATP released from astrocytes is the source of purinergic drive to CO_2_/pH-sensitive RTN neurons (Mulkey et al., 2006; Gourine et al., 2010; Huckstepp et al., 2010b; Wenker et al., 2010; Kasymov et al., 2013). Interestingly, work by Wenker et al. (2012) showed that CBX in concentrations that block both hemichannels and pannexons, significantly reduced the CO_2_-induced firing rate in RTN neurons. Given that hypercapnic stimulation of RTN neurons persisted in the absence of extracellular Ca^2+^, the authors proposed that CO_2_/pH-induced ATP release at the RTN relies on astroglial hemichannels rather than neuronal exocytosis (Wenker et al., 2012). Further molecular [e.g., tissue-specific inducible knockouts (KO)] and pharmacological (e.g., mimetic peptides) experiments are necessary to entirely understand the participation of astrocytes hemichannels and pannexons in central chemoreception and breathing control.

Besides breathing adaptations during high CO_2_ conditions, ventilation also needs to increase in circumstances of acute or chronic exposure to low levels of O_2_, thus coping with tissue O_2_ demands. This ventilatory reflex bases almost exclusively on the activation of peripheral but not central chemoreceptors. The major arterial peripheral chemoreceptors are the carotid bodies (CBs). Located bilaterally at the carotid bifurcation region, they embrace a polymodal ability to sense several stimuli, including a high sensitivity to changes in arterial O_2_ tension (Gonzalez et al., 1994). The CBs are organized in clusters of chemosensory units composed of chemoreceptor type I cells innervated by sensory terminals of the carotid sinus nerve, the whole being enwrapped by glial-like type II cells (Chen and Yates, 1984). Chemical synapses represent the major synaptic transmission route within the CBs and many transmitters have already been described in this system (Iturriaga and Alcayaga, 2004; Nurse, 2014). Although the precise mechanism underpinning CB chemoreception remains ignored, there is a consensus that chemical stimuli (hypoxia, acidity or hypercapnia) depolarize type I chemoreceptor cells, resulting in the Ca^2+^/vesicular-dependent release of ATP and subsequent firing in sensory terminals (Gonzalez et al., 1994; Nurse, 2010). The latter elicits a chemoreflex response that elevates ventilation and restores blood O_2_ and CO_2_ tension, as well as pH levels (Eyzaguirre and Zapata, 1984; Gonzalez et al., 1994; Iturriaga and Alcayaga, 2004; Nurse, 2010).

A series of studies have pointed out a possible role of Panx1 channels and purinergic signaling in peripheral CB-mediated chemoreception. It is known that ATP released at the synaptic cleft stimulates paracrine P2Y_2_ receptors of adjacent glial-like type II cells, resulting in [Ca^2+^]_i_ increase (Xu et al., 2003). A few years ago, Zhang et al. (2012) demonstrated that P2Y_2_ receptor-dependent rise in [Ca^2+^]_i_ is associated to prolonged depolarization and non-selective currents sensitive to CBX in concentrations that block in a greater degree Panx1 channels. The latter findings are consistent with the fact that P2Y receptor activation and consequent increase in [Ca^2+^]_i_ result in the opening of Panx1 channels (Locovei et al., 2006). Similarly, a follow-up work showed that angiotensin II acting on AT_1_ receptors in glial-like type II cells triggers Panx1 channel opening and consequently ATP release (Murali et al., 2014). ATP from both, glial and chemoreceptor sources, is hydrolyzed extracellularly into adenosine which enhances chemoreceptors depolarization through A_2A_ receptors, leading to more release of ATP (Murali and Nurse, 2016). These data suggest that activation of Panx1 channels in glial-like type II cells during chemotransduction contributes a positive feedback mechanism to potentiate the stimulus-evoked excitatory purinergic transmission between CB chemoreceptors and sensorial endings. This novel and interesting hypothesis deserves further investigation in order to elucidate whether it actually takes place *in vivo*.

## Neuroinflammation, Persistent Opening of Hemichannels/Pannexons and Synaptic Excitotoxicity

So far, we have discussed the multiple synaptic roles that hemichannels could perform at the normal nervous system. Nonetheless, another issue that has received increasing attention is how hemichannels, under certain pathophysiological scenarios, may favor brain disease progression. Hemichannels could be deleterious by (i) releasing excitotoxic levels of transmitters (e.g., ATP and glutamate), (ii) disturbing [Ca^2+^]_i_ handling or (iii) altering cytoplasmic ionic and osmotic balance (Vicario et al., 2017). A keystone underlying this phenomenon came from the long-lasting production of inflammatory signals as a result of the impaired operation of the brain innate and adaptive immune system (Kim et al., 2016). Indeed, acute and chronic neurodegenerative conditions are often accompanied of neuroinflammation, which is characterized by reactive gliosis, release of inflammatory agents (chemokines, cytokines, NO, reactive oxygen and nitrogen species [ROS/RNS]) and in special circumstances of BBB breakdown and consequent entry of circulating immune cells (Becher et al., 2017). Reactive gliosis encompasses a sequential and multistage conserved microglial and astroglial response that reduces acute injury, recovering the homeostasis and confining brain damage (Kettenmann et al., 2011; Pekny and Pekna, 2014). However, during intense and persistent brain injury, both microglia and astrocytes may become a vast source of detrimental molecules rather than contributing to protect and control cell dysfunction. There is plenty of data demonstrating the detrimental effects of inflammation on glial cells and neuronal function (Giaume et al., 2013), but how hemichannels might participate in this process is just beginning to be understood.

Most evidence points to persistent hemichannel opening as a crucial event in the genesis and progression of glial cell dysfunction (Giaume et al., 2013). Diverse inflammatory mediators (e.g., cytokines, NO and ROS) are recognized inducers of hemichannel and pannexon activity in astrocytes and microglia (Retamal et al., 2006, 2007a; Takeuchi et al., 2006; Abudara et al., 2015; Avendano et al., 2015; Gajardo-Gomez et al., 2017). Early studies by Takeuchi et al. (2006) revealed that TNF-α induces glutamate efflux via microglial Cx32 hemichannels, while similar findings have been observed in human microglial CHME-5 cells (Shaikh et al., 2012). TNF-α in combination with IFN-γ evokes the opening of Cx43 hemichannels and Panx1 channels in EOC20 microglial cells (Sáez et al., 2013b). In the same line, the combination of TNF-α and IL-1β elevates the activity of astroglial Cx43 hemichannels by a pathway relying on p38 MAP kinase signaling and further NO generation (Retamal et al., 2007a; Abudara et al., 2015). These findings seem to support the idea that glial activation triggered by pathological agents may evoke the aperture of hemichannels and pannexons through the autocrine release of cytokines and subsequent activation of multiple downstream inflammatory agents such as NO, prostaglandins, ATP, and ROS. In agreement with this assumption, simultaneous neutralization of TNF-α and IL-1β with IL-1ra and sTNF-aR1, completely prevents the hemichannel and pannexon opening evoked by prenatal LPS exposure and amyloid-β peptide (Aβ) treatment (Avendano et al., 2015; Gajardo-Gomez et al., 2017). Moreover, the stimulation of iNOS and COX_2_, as well as the elevated levels of [Ca^2+^]_i_ and NO, sustain the Panx1 channel-dependent release of ATP in LPS-treated microglia (Orellana et al., 2013), whereas NO-dependent Cx43 S-nitrosylation is critical in the opening of astroglial hemichannels induced by ROS (Retamal et al., 2006). Certainly, the activation of these cascades has been associated to glial hemichannel/pannexon activity in several disease contexts, such high cholesterol diet (Orellana et al., 2014), Aβ treatment (Orellana et al., 2011b; Gajardo-Gomez et al., 2017), restraint stress (Orellana et al., 2015), prenatal LPS exposure (Avendano et al., 2015), spinal cord injury (Garre et al., 2016), Alzheimer’s disease (Yi et al., 2016) and Niemann-Pick type C disease (Sáez et al., 2013a).

A pivotal feature in eliciting glial hemichannel activity relates to the immunomodulatory crosstalk that glial cells exert each other. For instance, microglia stimulated by pathological agents produce high levels of TNF-α and IL-1β, resulting in prominent *in vitro* and *ex vivo* astroglial Cx43 hemichannel activity (Retamal et al., 2007a; Abudara et al., 2015). Remarkably, microglia-mediated astroglial Cx43 hemichannel opening triggers Ca^2+^ entry and subsequent glutamate efflux, which disturbs hippocampal synaptic function (Abudara et al., 2015). At the other end, the release of ATP through astroglial Cx43 hemichannels and/or Panx1 channels (Braet et al., 2003; Iglesias et al., 2009; Garré et al., 2010) embraces a central pathway by which astrocytes govern microglial function (Verderio and Matteoli, 2001; Schipke et al., 2002). ATP-mediated opening of Cx43 hemichannels and Panx1 channels could evoke Ca^2+^-dependent release of ATP in microglia via the stimulation of P2X_7_ receptors (Bernier et al., 2012; Sáez et al., 2013b). Although opening of P2X_7_ receptors rise [Ca^2+^]_i_ (Baroja-Mazo et al., 2013), a well-known condition increasing hemichannel and pannexon activity (Locovei et al., 2006; De Bock et al., 2012b); ATP release *via* Panx1 channels likely involves protein-protein interactions between Panx1 and P2X_7_ receptors (Locovei et al., 2007). In fact, P2X_7_ receptor-mediated activity of Panx1 channels has been associated to the release of IL-1β by a pathway involving the activation of the inflammasome (Pelegrin and Surprenant, 2006; Kanneganti et al., 2007). In this line, the opening of Panx1 channels in neurons and astrocytes causes caspase-1 activation along with stimulation of different elements of the multiprotein inflammasome complex, such as the P2X_7_ receptor (Silverman et al., 2009; Murphy et al., 2012; Minkiewicz et al., 2013).

Because hemichannels formed by Cx26 and Cx43 are permeable to Ca^2+^ (Schalper et al., 2010; De Bock et al., 2012b; Fiori et al., 2012) and their opening is regulated by [Ca^2+^]_i_ (De Bock et al., 2012a; Meunier et al., 2017), one would expect that inflammation could increase glial hemichannel activity, resulting in abnormal Ca^2+^ dynamics and altered [Ca^2+^]_i_ homeostasis. Ca^2+^ signaling is fundamental for maintaining biological processes that govern glial survival and glia-to-neuron communication such as mitochondrial metabolism, antioxidant defense, metabolic substrate production and gliotransmitter release (Verkhratsky et al., 2012). In this context, impairment of [Ca^2+^]_i_ homeostasis linked to glial hemichannel opening could be critical in the possible vicious cycle underlying glial dysfunction during neuroinflammation (Agulhon et al., 2012). Accordingly, during inflammatory conditions, including treatments with LPS, IL-1β/TNF-α or amyloid-β peptide (Aβ), hemichannel opening has been associated with disturbed [Ca^2+^]_i_ dynamics and reactive gliosis (Orellana et al., 2010; Sáez et al., 2013a; Abudara et al., 2015; Avendano et al., 2015; Rovegno et al., 2015; Gajardo-Gomez et al., 2017). How might hemichannels trigger glial cell death? Besides to impair [Ca^2+^]_i_ dynamics, Ca^2+^ entry via hemichannels may cause Ca^2+^ overload, which could lead to free radical formation, lipid peroxidation and plasma membrane damage. Furthermore, osmotic and ionic imbalance evoked by the prolonged influx of Na^+^ and Cl^-^ through hemichannels also could lead to subsequent cell swelling and plasma membrane breakdown.

**FIGURE 4:**
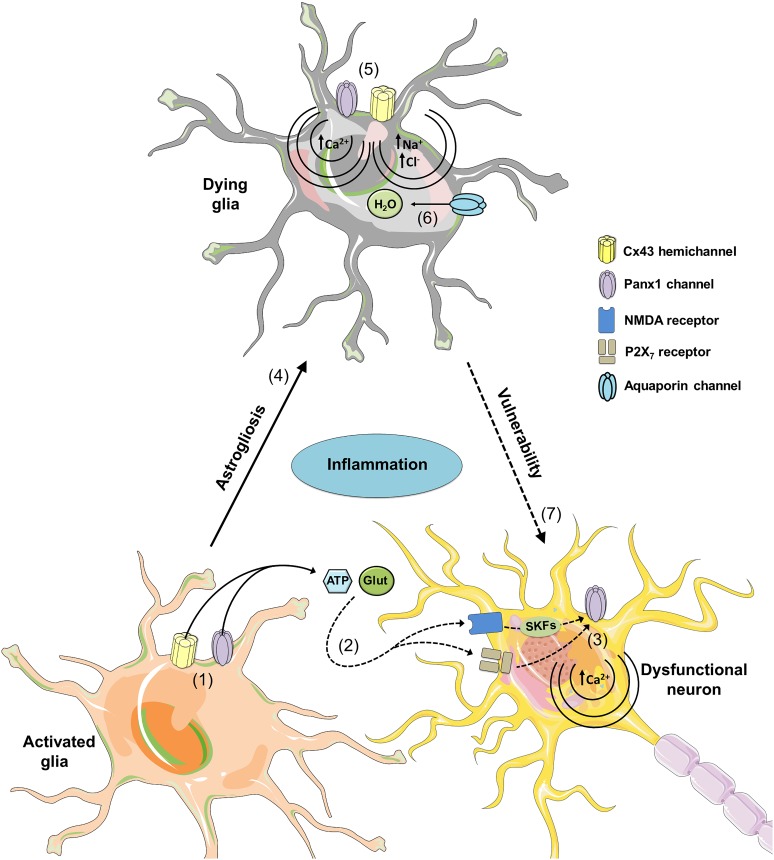
Possible roles of glial hemichannels and pannexons during neuroinflammation. At early stages of different neurodegenerative diseases, increased inflammation activates glial Cx43 hemichannels and Panx1 channels (1), resulting in the release of gliotransmitters (ATP and glutamate) and further stimulation of NMDA and P2X_7_ receptors in neurons (2). NMDA and P2X_7_ receptor activation possibly increases the opening of neuronal Panx1 channels through phosphorylation of Panx1 by Src family kinases (SFKs) and direct protein-to-protein interactions, respectively (3). The latter could affect [Ca^2+^]_i_ homeostasis leading to cell damage and further death. Uncontrolled activation of glial cells may result in reactive gliosis and subsequent damage by a mechanism that implicates the opening of hemichannels and pannexons (4). Specifically, permanent opening of Cx43 hemichannels and Panx1 channels could cause cell damage by different mechanisms. At one end, Ca^2+^ entry via Cx43 hemichannels or Panx1 channels may activate phospholipase A_2_ with the subsequent generation of arachidonic acid and activation of cyclooxygenase/lipoxygenase pathways, which consequently leads to elevated levels of free radicals, lipid peroxidation and plasma membrane breakdown (5). At the other end, Na^+^ and Cl^-^ entry via Cx43 hemichannels or Panx1 channels may induce cell swelling due to an increased influx of H_2_O via aquaporins (6). Certainly, given that glial cells provide support to neurons; glial cell damage associated with hemichannel/pannexon opening could indirectly increase neuronal susceptibility and vulnerability to the homeostatic imbalance occurring during neurodegeneration.

How might inflammation-induced glial hemichannel opening impair neuronal function and survival? At this regard, it is possible to hypothesize that hemichannel-mediated glial dysfunction may affect neuronal function and survival by two mechanisms: (1) by making neurons more susceptible to damage evoked by neuroinflammation itself and (2) by altering glia-to-neuron gliotransmission (Figure 4). Because neurons require proper metabolic, antioxidant and trophic support from glial cells; likely their damage linked to hemichannel opening might collaterally enhance neuronal vulnerability to inflammation (Figure 4). Indeed, during well-known inflammatory conditions, including focal ischemia and traumatic brain injury, glial demise precedes delayed neuronal death, suggesting that glial survival is crucial for neuroprotection (Liu et al., 1999; Zhao et al., 2003). Although there is compelling evidence indicating that persistent hemichannel opening leads to glial cell death (Contreras et al., 2002; Orellana et al., 2010; Okuda et al., 2013; Rovegno et al., 2015), it is ignored whether this pathway might account for an important portion of the neuronal death in the inflamed brain or whether it occurs only in specialized circumstances.

On the other hand, the persistent opening of glial hemichannels triggered by inflammation may induce the uncontrolled release of gliotransmitters (e.g., ATP, glutamate and D-serine) that might be excitotoxic for neurons (Figure 4). According to this idea, astrocytes or microglia stimulated with Aβ release high amounts of glutamate and ATP via the opening of Cx43 hemichannels and pannexons, which results toxic for hippocampal and cortical neurons (Orellana et al., 2011a). A later study revealed that astrocytes pre-incubated with conditioned media from Aβ-stimulated microglia, release excitotoxic levels of glutamate and ATP through Cx43 hemichannels when treated with hypoxia in high glucose (Orellana et al., 2011b). Similar hemichannel-mediated excitotoxicity has been found in glial cells stimulated with TNF-α (Takeuchi et al., 2006), as well as in animal models of Alzheimer’s disease, ischemia or brain injury (Danesh-Meyer et al., 2012; Ishii et al., 2013; Yi et al., 2016; Gangoso et al., 2017). Substantial evidence has shown that hemichannel-dependent release of glutamate and ATP diminishes neuronal survival through the stimulation of neuronal NMDA/P2X_7_ receptors and Panx1 channels (Orellana et al., 2011a; Avendano et al., 2015). How glutamate and ATP impact neuronal hemichannel function and survival? Current data point out that neurons express functional Panx1 channels (Thompson et al., 2006, 2008) and their opening, as previously noted, could take place by protein–protein interactions with activated P2X_7_ receptors (Locovei et al., 2007) or via raising of [Ca^2+^]_i_ (Locovei et al., 2006). In addition, the interaction of NMDA receptors with Src family kinases causes phosphorylation of Panx1 C-terminus and subsequent pannexon activity (Weilinger et al., 2012).

## Concluding Remarks

Theoretically, synaptic-mediated changes in the number of functional hemichannels and pannexons at the glial cell membrane could operate in timescales ranging from seconds to hours and through a wide variety of mechanisms. Of particular interest for future studies are regulations related to gating properties, changes in trafficking of preformed channels or in the synthesis rate of *de novo* channels. In addition, alterations in the GJC/hemichannel ratio, as well as in the profile of contributing channels with different permeability properties to the synaptic cleft could, in theory, impact synaptic transmission. The temporal course of those mechanisms (milliseconds to hours) is physiologically relevant since it will set the temporal outcome for shaping either short-term (milliseconds to a few minutes) or long-term (minutes to hours) plasticity. In this scenario, glial undocked hemichannels and pannexons emerge as alternative non-vesicular pathways for gliotransmission to dynamically regulate neuro-glial crosstalk, neuronal networks, synaptic plasticity and high brain functions under physiological circumstances. Both hemichannels and pannexons provide a mechanism to adjust the gain of synaptic transmission and reshape the neural outcome in either resting or stimulated conditions. However, in pathological situations, alterations in hemichannel/pannexon function may result in inflammatory signaling that impairs glial survival and likely results in an excitotoxic mechanism that alters synaptic transmission and plasticity. As the function of these channels differs among physiological or pathological contexts, their signaling may act as a double edge sword facilitating the synaptic transmission or perpetuating synaptic impairment and cellular damage. Albeit progress has been done in order to deepen our knowledge about the role of hemichannels and pannexons during neurotransmission, supplementary research is required to assess their contribution *in vivo*.

## Author Contributions

VA, MAR, RDR, and JAO conceived and designed the major ideas developed in the manuscript, and wrote and edited the manuscript. JAO designed the figures. All authors read and approved the final manuscript.

## Conflict of Interest Statement

The authors declare that the research was conducted in the absence of any commercial or financial relationships that could be construed as a potential conflict of interest.
